# Association of physical activity and positive thinking with global sleep quality

**DOI:** 10.1038/s41598-022-07687-2

**Published:** 2022-03-07

**Authors:** Morgan Chen, Zhonghui He, Zhanjia Zhang, Weiyun Chen

**Affiliations:** 1 School of Kinesiology, University of Michigan, 830 N University Avenue, Ann Arbor, MI 48109 USA; 2 Division of Physical Education, Peking University, Beijing, China

**Keywords:** Risk factors, Psychology

## Abstract

This study examined the association of different intensity levels of physical activity and positive thinking with a global sleep quality among college students. The research question was: to what degree were the different intensity levels of physical activity and positive thinking significantly associated with the global sleep quality among college students? We recruited students, who enrolled in regular physical education classes during a fall semester at a major public university. 553 students signed the consent form and indicated their voluntary participation in this study. The final data set for analysis consisted of 403 college students with the mean age of 19.01 years ± 1.559 years (217 males vs. 186 females) based on the results of data screening. They completed three questionnaires: *International Physical Activity Questionnaire (IPAQ), Pittsburgh Sleep Quality Index (PSQI), and Positive Thinking Scale (PTS)* during a regular physical education class. The multiple regression model revealed that vigorous-intensity physical activity, positive thinking, and negative thinking were collectively and individually associated with the Global PSQI sleep quality (*F* = 19.389, *p* = .000), explaining 12.8% of the total variance in the Global PSQI sleep quality for the total sample. College students’ engaging in vigorous intensity level of physical activity, and having a good level of positive thinking and a low level of negative thinking were both collectively and individually linked to the Global PSQI sleep quality.

## Introduction

The National Sleep Foundation recommends 7 to 9 h of sleep, with 6 h as a minimum and 10 to 11 as the appropriate maximum to achieve a good quality of sleep^1^. Further recommendations for improving quality of sleep include exercising regularly, avoiding alcoholic drinks close to bedtime, and participating in relaxing activities before bed^1^. However, college students are one of the most sleep-deprived populations^2^. Up to 60% of college students suffer from sleep deprivation and poor sleep quality associated with sleep disturbances^1,2^ Sleep deprivation is a failure to obtain adequate amounts of sleep. Sleep deficiency is the presence of sleep deprivation, disrupted sleep cycles, or a sleeping disorder^3^. College students tend to give up their sleep hours for longer studying time, but this action may be counterproductive. A lack of sleep likely impedes their learning efficiency in the short-term and negatively influences their health status for the long-term^4^. Poor sleep quality is associated with poor health conditions, absenteeism from school, and increased risk for psychiatric disorders, including depression and generalized anxiety disorders^5^. Sleep is imperative to the overall survival and fitness of all organisms and positively influences energy saving and restoration, immune function, and emotional well-being^6,7^.

Participation in physical activity can improve sleep quality and decrease symptoms of sleep disorders^8^. The 2018 Physical Activity Guidelines for Americans^9^ recommend that adults should participate in at least 150 to 300 min of moderate-intensity physical activity a week, or 75 to 150 min of vigorous-intensity physical activity, or an equivalent combination of moderate and vigorous-intensity physical activity a week. Unfortunately, a majority of college students do not meet the recommended amount of physical activity^10^. Approximately 6 out of 10 students are engaging in less than 3 days of vigorous-intensity (20 min or more) or moderate-intensity (30 min or more) of physical activity per week^10^.

It is empirically evident that intensity levels of physical activity impact sleep quality^11–16^. In a study of effects of a 6-month physical activity intervention on sleep and mood, the intervention participants (n = 20) aged 40 years or older significantly reduced insomnia symptoms, increased quality of sleep, and elevated mood after completing the supervised ≥ 150 min of moderate- to vigorous-intensity physical activity per week, compared to 21 control participants^11^. Another study examined effects of a 12-week Pilates intervention program on sleep quality among 99 sedentary middle-aged participants^12^. The results showed that the intervention participants, who attended two one-hour Pilates intervention per week, significantly reduced sleep latency and increased overall sleep quality as well as increased physical activity over time, compared to the control group^12^. A meta-analysis review of 66 studies examined the effects of “acute” exercise and “regular” exercise on sleep quality^13^. Acute exercise refers to participants engaging in less than 30 min of moderate intensity exercises 3 days per week for at least 3 months; regular exercise means that participants engaged in greater than 30 min of moderate intensity exercises 3 days per week for at least 3 months. The review found that acute exercise produced small positive effects on total sleep time, slow wave sleep, and sleep onset latency^13^. In contrast, regular exercise generated moderate to large positive effects on seven components of sleep on Pittsburgh Sleep Quality Index (PSQI)^13^. In addition, in a study of 434 young adults with an average age of 17 and 258 of these being athletes^14^, the results found that adolescent athletes, who engaged in vigorous exercises regularly, reported better sleep patterns and lower scores for depressive symptoms compared to the control participants^14^. However, the relationship between intensity levels of physical activity and sleep quality still remains largely unexplored for college student population.

Better sleep quality can help individuals be revitalized and wake up with feelings of “refreshed.” There is an increased interest in examining whether a better sleep can generate increased positive thoughts. Positive thinking is an individual’s belief in one’s ability to overcome barriers for making behavioral changes and achieving self-determined goals^15,16^. Positive thinking primarily focuses on people’s positive views of themselves and their positive thoughts on important aspects of life^15–17^. Positive thinking moderates how people respond to stressful events and define meaning in life^17^. People with high levels of positive thinking report a significant positive relationship between event stressfulness and meaning in life^17^. A recent longitudinal study of 307 adults cohort aged 33–65 years found that poor sleep resulted in greater attention to negative emotions, whereas good sleep was associated with better emotional regulation and greater positive emotions^18^. However, there is a dearth of study examining to extent to which positive thinking is associated with sleep quality among college students.

To the best of our knowledge, little is known about the degree to which different intensity levels of physical activity and positive thinking are related to global sleep quality among college students. Thus, the purpose of this study was to determine if different intensity levels of physical activity and positive thinking were associated with global sleep quality among college students.

## Methods

### Research participants

In this study, we targeted to recruit students, who enrolled in regular physical education classes during a fall semester at a major public university in Beijing, China. The second author is a physical education (PE) teacher at the major university where the study took place. She was trained in the recruitment procedures by the senior author and was responsible for recruitment of participants. She sent an invitation letter indicating the study purpose, design, benefits, and protocols to her colleagues. Upon receiving 13 PE teachers’ indication of willing to help the recruitment, the second author trained them in the protocols for recruitment and administration of the questionnaire during a regular PE program meeting. One week later, the trained PE teachers followed the protocols to help recruit their students to participate in this study by: (a) explaining the study purpose, voluntary participation, confidentiality assurance, and no penalty for choosing not completion of the questionnaires during a regular PE class; (b) informing students can participate upon returning their signed consent forms; (c) distributing the consent form to their students during a regular PE class; and (d) collecting the signed consent forms from the students one week later.

As a result of recruitment, 553 students who were enrolled in one of the physical education classes, signed the consent form and indicated their voluntary participation in this study. Each PE class offered one 90-min PE content-specific lesson per week over the course of the semester. Table 1 presents the participants’ demographic information including age, sex, years of college, and types of PE class. The final set data consisted of 403 participants due to deletion of missing cases and outliers described in the data analysis below. The study protocols (HUM00102146) were approved by the University Institutional Review Board-Health Sciences and Behavioral Sciences (IRB-HSBS).

**Table 1:** Participants’ demographic information.

Variables	n	%	Mean	SD
**Age**				
Total sample			19.01	1.559
Males			18.92	1.788
Females			19.11	1.236
**Gender**				
Males	217	53.8		
Females	186	46.2		
**Grade**				
Freshman	27	6.7		
Sophomore	75	18.6		
Junior	119	29.5		
Senior	182	45.2		
**PE classes**				
Aerobic exercises	50	12.4		
Badminton	121	30		
Basketball	13	3.2		
Strength and conditioning	158	39.2		
Swimming	32	7.9		
Tai Chi	13	3.2		
Tennis	16	4.0		

### Data collection

The trained PE teachers administered the questionnaires to the students in a regular PE class during the second or third week of October. Prior to completing the questionnaires, participants were asked to fill out their demographic information, including age, sex, years of college, and a PE class taken. Then, participants were asked to follow the directions to complete each of three questionnaires.

#### Sleep

The students completed the *Pittsburgh Sleep Quality Index (PSQI)* which was designed to measure a quality and patterns of sleep in adults^19^. The PSQI consists of four broad questions (i.e. How long does it take you to fall asleep at night?) and 15 specific questions on a 4-point rating scale to measure seven components: subjective sleep quality, sleep latency, sleep duration, habitual sleep efficiency, sleep disturbances, use of sleep medications, and daytime dysfunction. Participants were asked to recall and answer each question based on the past month. The seven component scores are added together to obtain the Global PSQI score. The Global PSQI score of 5 or higher indicates poor sleep quality. The PSQI has been proven to be a reliable and valid measure to assess a quality of sleep^19^. In our study, the Global PSQI has a Cronbach’s alpha of 0.700, indicating an acceptable internal consistency.

#### Physical activity

*International Physical Activity Questionnaire-Short Form (IPAQ-S)*^20,21^ was designed to measure an individual’s engagement of various intensity levels of physical activity and the amount of sitting time in their daily lives. Participants were asked to recall and answer each question based on the last 7 days. Questions consists of vigorous- and moderate-intensity physical activity, walking, and sitting (i.e. During the last 7 days, on how many days did you do vigorous physical activities like heavy lifting, digging, aerobics, or fast bicycling?). The specific types of activities were assessed for frequency (days per week) and duration (time per day). According to the guidelines for data processing and analysis of the IPAQ-S (2015)^20^, a specific formula with assigned Metabolic Equivalent for Task (MET) values was used to calculate the MET-minutes/week score for walking, moderate-intensity physical activity, vigorous-intensity physical activity, and a total weekly physical activity. For example, the formulas are: the Walking MET-min/week = 3.3 × walking min × walking ‘days’; Moderate MET-min/week = 4.0 × moderate-intensity activity min × moderate days; Vigorous MET-min/week = 8.0 × vigorous-intensity activity min × vigorous-intensity days; a total physical activity MET-min/week can be computed as the sum of Walking + Moderate + Vigorous MET-min/week scores^20^. The IPAQ-S has proven to be a reliable and valid measure of physical activity^22^. In this study, the IPAQ has a Cronbach’s alpha of 0.702, indicating an acceptable internal consistency.

#### Positive thinking

The students completed the 22-item *Positive Thinking Scale (PTS) on a “yes–no” format*^16^. 11 items represent positive thoughts and perceptions, while other 11 items represent low negative thinking. Answers to the questions were based on their feelings in the past month. One point was awarded for each “yes” response to the positive items and one point was awarded for each “no” response to the negative items. After reversing the scores of negative items, each score of the 22 items was added up to generate a score range of 0–22, with the higher score representing higher levels of positive thoughts and the tendency to think in positive ways^16^. In this study, the PTS has a Cronbach’s alpha of 0.873, indicating an acceptable internal consistency.

### Data analysis

Prior to conducting statistical analyses, three steps were used to screen the data set consisting of three questionnaires. First, each participant’s responses to the questions of PSQI were checked. 15 cases were deleted using a listwise deletion due to missing values of PSQI. Then, 30 outliers of the Global PSQI score were identified by using the SPSS_Explore method. They were deleted out of the data set. Second, the same methods as above were used to check each participant’s responses to the questions of PTS. No case was deleted because of no missing values or outliers of PTS. Third, each participant’s responses to the IPAQ-S were screened using the same methods as above. As a result, 92 cases were deleted due to missing values of the IPAQ-S. Next, 13 outliers of the IPAQ-S were detected. The data set consisting of 403 cases was used for a final data analysis in this study.

Descriptive statistics for each sub-scale and total scale of the PSQI, IPAQ-S, and PTS were computed for the total sample and by gender. Skewness and kurtosis for the Global PSQI score, the total scale score of PTS, and the total MET score of IPAQ-S were checked for normality. According to the normality criteria for skewness between − 2 to 2 and kurtosis between − 7 to 7 indicating normality^23^, the results showed that the skewness of 0.442 and Kurtosis of − 0.356 for Global PSQI score, the skewness of 0.937 and Kurtosis of 0.154 for the total MET score, and the skewness of − 0.376 and Kurtosis of − 0.781 for the total PTS score.

Bivariate correlation coefficients between the Global PSQI score and each sub-scale and the total scale of the IPAQ-S and the total scale of the PTS were computed to determine if there is a correlation between them. Then, a multiple R-Squared linear regression model with a stepwise method was conducted to determine the extent to which different intensity levels of physical activity and the PTS total scale were associated with the Global PSQI score for the total sample, males, and females separately. Subsequently, standardized multiple regression coefficients were analyzed to assess the relative importance of each independent variable individually predicting of the Global PSQI sleep quality for the total sample, males, and females separately. All statistics methods were conducted with IBM SPSS 27. A significance level of *p* < 0.05 was set for all statistical methods.

### Ethical approval

This study (HUM00102146) protocols were approved by the University Institutional Review Board-Health Sciences and Behavioral Sciences (IRB-HSBS). All methods were performed in accordance with the relevant guidelines and regulations.

### Consent to participate

Informed consent was obtained from all individual participants included in the study.

### Consent to publish

All authors have seen and approved the final version of the manuscript being submitted. They warrant that the article is the authors' original work, hasn't received prior publication and isn't under consideration for publication elsewhere. Authors are responsible for correctness of the statements provided in the manuscript. See also Authorship Principles. The Editor-in-Chief reserves the right to reject submissions that do not meet the guidelines described in this section.

## Results

### Preliminary analysis

#### Sleep quality

Table 2 presents descriptive statistics of the seven components of sleep quality and the Global PSQI score for the total sample and by gender. Based on the PSQI scoring coding system, the lower score of each component, the better level of sleep quality. As seen the Table 2, the mean score of Subjective Sleep Quality was 0.82, indicating that students had fairly good level of subjective sleep quality based on the 4-point rating scale (0 = very good, 1 = fairly good, 2 = fairly bad, and 3 = very bad). The mean score of Sleep Latency was 1.02, indicating that students took 16–30 min to fall asleep each night based on the 4-point rating scale (0 ≤ 15 min, 1 = 16–30 min, 2 = 31–60 min, and 3 ≥ 60 min). The mean score of Sleep Duration was 0.89, showing that students had approximate 6–7 h of sleep at night based on the 4-point rating scale (0 ≥ 7 h, 1 = 6–7 h, 2 = 5–6 h, and 3 ≤ 5 h). Likewise, the mean score of Habitual Sleep Efficiency was 0.06, indicating that students had a good level of sleep efficiency based on the 4-point rating scale (0 ≥ 85%, 1 = 75–84%, 2 = 65–74%, and 3 ≤ 65%), about 80% efficient. The mean score of Sleep Disturbances was 0.98 based on the 4-point rating scale (0 = 0, 1 = 1–9, 2 = 10–18, and 3 = 19–27), indicating students did not experience sleep disturbances often. Similarly, the mean score of Use of Sleep Medication was 0.06, indicating that the students rarely used sleep medication. The mean score of Daytime Dysfunction was 1.06 based on the 4-point rating scale (0 = not during the past month, 1 = less than once a week, 2 = once or twice a week, and 3 = three or more times a week), indicating the students less often experienced daytime dysfunction. Lastly, the mean score of the Global PSQI was 4.89, indicating that students’ quality of sleep was not poor on average because a total score of “5” or greater is indicative of poor sleep. Furthermore, both male and female students had a similar level of sleep quality in terms of all seven components and the Global PSQI.

**Table 2:** Means and standard deviations for participants’ PSQI sub-scale and total scores.

Variables	$\overline{X }$			*SD*		
	Total	Males	Females	Total	Males	Females
Subjective sleep quality	0.82	0.78	0.86	0.630	0.627	0.634
Sleep latency	1.02	1.03	1.01	0.788	0.813	0.760
Sleep duration	0.89	0.92	0.87	0.734	0.725	0.746
Habitual sleep efficiency	0.06	0.05	0.07	0.232	0.210	0.256
Sleep disturbances	0.98	0.97	1.0	0.411	0.384	0.441
Use of sleep medications	0.06	0.06	0.08	0.354	0.283	0.422
Daytime dysfunction	1.06	1.04	1.09	0.789	0.765	0.817
Global PSQI score	4.89	4.85	4.97	1.936	1.907	1.974

#### Physical activity

Table 3 shows the means and standard deviations of the four variables in the IPAQ-S. According to the guidelines for IPAQ scoring, three levels of activity classifications: health enhancing physical activity (HEPA) active, minimally active, and inactive were generated. To achieve HEPA active: an individual is required to accumulate at least 1500 MET-min/week in vigorous activity or achieve a minimum of at least 3000 MET-min/week in the combination of walking, moderate, and vigorous activity. To achieve minimally active, a person must achieve a minimum of at least 600 MET-min/week in the combination of the three activities. For the inactive, a person does not meet the criteria for the HEPA category or the minimally active category. As seen in Table 3, the mean score of the total MET-min/week of physical activity was 1977.34. The results showed that the students’ physical activity level, on average, was in the minimally active category. Further, analysis of the mean scores of the three activities indicated low levels of walking, moderate, and vigorous activity in which the students were engaged during the past 7 days. Although both male and female students did not reach the HEPA active, the results of the t-tests revealed that male students had a significantly higher level of Vigorous PA, Moderate PA, and Total weekly PA than did female students (*t* = 2.857, *p* = 0.004; *t* = 2.093, *p* = 0.037; *t* = 2.969, *p* = 0.003).

**Table 3:** Means and standard deviations of IPAQ sub-scale and total scores (MET-min/week).

Variables	$\overline{X }$			*SD*		
	Total	Males	Females	Total	Males	Females
Vigorous PA	770.74	888.89	632.04	910.44	938.91	857.77
Moderate PA	590.19	663.04	504.28	773.38	840.04	678.77
Walking	625.07	623.27	627.16	700.78	703.37	699.65
Total PA	1977.34	2171.11	1751.27	1437.76	1476.68	1360.33

#### Positive thinking

Table 4 presents the means and standard deviations of the variables in the PTS. As shown in Table 4, the mean score of the total scale was 14.96 based on the possible range of score: 0–22. Accordingly, the higher score represents the persons’ tendency to think about themselves, others, and important aspects of life in positive ways. The results showed that students’ responses, on average, reflected a tendency to think positively. Furthermore, both male and female students had similar scores in Positive Thinking, Negative Thinking sub-scales and the total scale of PTS.

**Table 4:** Means and standard deviations of PTS sub-scale and total scores.

Variables	$\overline{X }$			*SD*		
	Total	Males	Females	Total	Males	Females
Positive thinking	9.58	9.64	9.76	1.933	1.808	1.528
Negative thinking	5.38	5.24	5.68	3.209	3.172	3.218
Total scale	14.96	14.88	15.44	4.567	4.466	4.306

### Relationship of physical activity and positive thinking with sleep

#### Bivariate correlation

Table 5 presents the results of bivariate (Pearson) correlations between Global PSQI and each variable of IPAQ-S and PTS. For the total sample, Global PSQI sleep quality was negatively and weakly correlated with Vigorous MET (*p* < 0.05) and moderately correlated with Positive Thinking, Negative Thinking, and Overall Positive Thinking scales at *p* < 0.01. For male students, Global PSQI sleep quality was negatively and weakly correlated with Total MET at *p* = 0.062 and moderately correlated with Positive Thinking, Negative Thinking, and Overall Positive Thinking at *p* < 0.01. For female students, Global PSQI sleep quality was weakly correlated with Walking MET at *p* = 0.056 and negative and moderately correlated with Positive Thinking, Negative Thinking, and Overall Positive Thinking at *p* < 0.01.

**Table 5:** Correlation values of total sleep and all variables.

Variables	Global PSQI score	*p*
**Total sample**		
Vigorous PA	− 0.108	0.031*
Moderate PA	− 0.036	0.468
Walking	− 0.010	0.836
Total PA	− 0.085	0.090
Positive thinking	− 0.339	0.000**
Negative thinking	− 0.305	0.000**
Overall positive thinking	− 0.351	0.000**
**Male students**		
Vigorous PA	− 0.120	0.078
Moderate PA	− 0.004	0.953
Walking	− 0.105	0.125
Total PA	− 0.127	0.062
Positive thinking	− 0.355	0.000**
Negative thinking	− 0.329	0.000**
Overall positive thinking	− 0.377	0.000**
**Female students**		
Vigorous PA	− 0.087	0.243
Moderate PA	− 0.076	0.308
Walking PA	0.140	0.056*
Total PA	− 0.025	0.738
Positive thinking	− 0.325	0.000**
Negative thinking	− 0.285	0.000**
Overall positive thinking	− 0.328	0.000**

*p < 0.05; **p < 0.01.

#### Multiple regression models

Based on the results of the significant correlations above, in the multiple R-squared linear regression model, independent variables were Vigorous PA, Positive Thinking, Negative Thinking, and Overall Positive Thinking, and a dependent variable was the Global PSQI quality sleep. Table 6 presents the results of a multiple R-squared linear regression model. The results revealed that Vigorous PA, Positive Thinking, and Negative Thinking were significantly associated with the Global PSQI score (*F* = 19.389, *p* = 0.000) with an exclusion of Overall Positive Thinking from the model. These independent variables collectively explained 12.8% of the total variance in Global PSQI sleep quality for the total sample. Subsequently, analysis of the standardized regression coefficients (*β*) revealed Vigorous PA, Positive Thinking, and Negative Thinking were significant individual predictors of the Global PSQI quality sleep (*t* = − 1.866, *p* = 0.06;* t* = − 4.160, *p* = 0.000; *t* = − 2.121, *p* = 0.035).

**Table 6:** Results of multiple R-squared regression model with sub-scales of IPAQ and PTS as predictors of global PSQI score.

Variables	*R*	*R*^2^	*F*	*p*	*Beta*	*t*	*p*
**Total sample**							
Model	0.358	0.128	19.389	0.000**			
Vigorous PA					− 0.089	− 1.866	0.063
Positive thinking					− 0.242	− 4.160	0.000**
Negative thinking					− 0.124	− 2.121	0.035*
Exclusion of overall positive thinking							
**Males**							
Model	0.393	0.155	12.977	0.000**			
Total PA					− 0.083	− 1.273	0.204
Positive thinking					− 0.258	− 3.316	0.001**
Negative thinking					− 0.149	− 1.892	0.060
Exclusion of overall positive thinking							
**Females**							
Model	0.326	0.106	7.223	0.000			
Walking					0.073	1.035	0.302
Positive thinking					− 0.214	− 2.424	0.017*
Negative thinking					− 0.128	− 1.457	0.147
Exclusion of overall positive thinking							

*p < 0.05; **p < 0.01.

Based on male students’ results of the significant correlations above, in the multiple R-squared linear regression model, independent variables were Total weekly PA, Positive Thinking, Negative Thinking, and Overall Positive Thinking, and a dependent variable was the Global PSQI quality sleep. As seen in Table 6, the results of regression model revealed that Total weekly PA, Positive Thinking, and Negative Thinking were significantly associated with the Global PSQI score (*F* = 12.977, *p* = 0.000) with an exclusion of Overall Positive Thinking from the model. The independent variables accounted for 15.5% of the variance in the Global PSQI sleep quality among male students. Subsequently, the standardized regression coefficients (*β*) revealed that Positive Thinking and Negative Thinking were significant individual predictors of the Global PSQI sleep quality (*β* = − 0.258, *t* = − 3.316, *p* = 0.001*; β* = − 0.149, *t* = − 1.892, *p* = 0.060).

Based on female students’ results of the significant correlations above, in the multiple R-squared linear regression model, independent variables were Walking, Positive Thinking, Negative Thinking, and Overall Positive Thinking, and a dependent variable was the Global PSQI quality sleep. As seen in Table 6, The results of the regression model for female students indicated that the four independent variables were significantly related to the Global PSQI sleep quality (*F* = 7.223, *p* = 0.000), explaining 10.6% of the total variance in the Global PSQI sleep quality among female students. Subsequently, the standardized regression coefficients (*β*) revealed that Positive Thinking (*β* = − 0.214 *t* = − 2.414, *p* = 0.017) was a significant individual predictor of the Global PSQI sleep quality for female students.

## Discussion

This study was central to examining the association of intensity levels of physical activity and positive thinking with the Global PQSI sleep quality among college students. In this study, the participating students’ global sleep quality was relatively good due to a good level of seven sleep components such as low sleep disturbances and good habitual sleep efficiency. However, the promising results of the study was inconsistent with the findings reported by a recent meta-analysis of 76 studies comprising of 112,939 college students in China^24^. The meta-analysis review found that sleep disturbances were prevalent among the Chinese university students^24^. Sleep disturbances are mainly linked to poor sleep quality^1^. The present study also showed that both male students and female students had similar good levels of sleep quality in terms of the seven components and the Global PSQI sleep quality. The results were supported by the meta-analysis study, which shows no gender differences occurred in sleep quality measured with PSQI^24^. Regarding physical activity participation, the students had an insufficient level of engaging in vigorous intensity, moderate intensity, and light intensity of physical activity. In other words, they were minimally active in all three levels of intensity. These results are consistent with previous studies that found a lack of engagement in moderate-to vigorous physical activity is prevailing among college students^10,24^. In this study, more concern is that female students were engaged in even lower level of vigorous intensity, moderate intensity, and total PA than their male counterparts. However, the promising result of this study is that both male and female students self-reported a high level of overall positive thinking and a low level of negative thinking.

As partially hypothesized, this study found that the college students’ engaging in vigorous intensity level of physical activity, having a good level of positive thinking and a low level of negative thinking were both collectively and individually associated with the Global PSQI sleep quality. The findings were in line with previous studies in which participants who meet the recommended physical activity guidelines were more likely to have better sleep quality^25–27^. A systematic review of six studies found that participants in an exercise intervention group indicated decreased Global PSQI score and significantly reduced sub-scale scores for sleep latency and medication use, compared to control groups^28^. Similarly, a study found that participation in moderate-to-vigorous physical activity during physical education classes promoted better sleep quality^29^. In addition, a recent study investigated the relationship between physical activity and sleep in adolescent students and found that males reported a shorter sleep latency score than females^29^. Contrary to the results^29^, the present study showed no significant gender differences in sleep latency and other components of sleep quality.

Given the positive relationship between vigorous-intensity physical activity and sleep quality, this study suggests that universities should consider implementing physical education classes as a part of course scheduling or require participation in intramural or club sports to further provide college students with a positive and successful living environment. A study found that only 18% of college students engage in physical activity 5 or more days a week and 23% report zero activity^34^. To meet students’ needs and interests in physical activity, university PE program should provide students with a variety of PE classes such as yoga, tai chi, meditation, running, team sports, and individual sports.

Further, the findings of this study were consistent with the study that found a strong association between positive thinking and quality of sleep^4^. Individuals who had positive thoughts were more likely to adhere to the recommended sleep hours^4^. Supporting the present results, studies of neural and behavioral reactivity reported a strong link between positive thinking and good sleep quality^3,4^. This study suggests that college students who have a positive mindset about themselves, others, and aspects of life tend to have a high quality of sleep, despite being students at the top university in China, who are facing highly competitive and demanding academic pressures. It is important to note that this positive relationship is reciprocal. Students with a positive outlook tend to sleep well. Students with adequate sleep and good quality of sleep are prone to having a positive mindset^30^. Likewise, a study conducted at another major public university found that people who had a purpose in life generally had good quality of sleep^31^. On the other hand, negative emotional reactivity has a significant interaction with sleep loss and mood disorders^32^. Insufficient sleep cannot be overlooked as a significant contributor to the growing high levels of mental health problems among university students^4^. Sleep deprivation is associated with increased reactivity to negative stimuli. Shorter bouts of sleep caused further attention on negative stimuli^33^.

The key implication of this study is that college students are at a critical age where health decisions can affect their future health and changes enforced at the university level could provide the necessary guidance to improve the relationship between physical activity, quality of sleep, and positive thinking. Further, universities should consider needs for greater flexibility in timing when registering for course offerings that could allow students to find a more consistent sleep cycle that can permit for naps during the day. It is seen that productivity and safety significantly dip shortly after noon, and young adults in many countries chose to take daytime naps at this time^35^. One study reported that even a very short nap (15–30 min), effectively offset diminished alertness, benefitted task performance, reduced sleepiness, improved mood, and reduced negative thoughts^35^.

The limitation of this study is related to a cross-sectional research design. Thus, no causational conclusions can be made. Future studies may consider using an experimental design to find the cause of physical activity and positive thinking on quality of sleep. Also, this study is limited in using a convenient and purposeful sample method to recruit the participants who were enrolled in physical education classes during a semester. To increase the generalizability of the study findings, future studies may expand recruitment inclusion criteria, including students who are not enrolled in physical education classes or not participated in any intramural or club sports to participate to make a sample more representative of the college student population. Further, nutrition could be a contributing factor that is not considered in this study. A study reports that foods such as milk products, fish, fruit, and vegetables may show sleep-promoting effects and certain dietary intake patterns may contribute to better quality of sleep^36^. Including measures of daily food intake, dietary habits, body composition (i.e., BMI or muscle mass) in future studies may help to build on this current study, examining both positive thoughts and a healthy diet as major contributors to good quality of sleep.

## Conclusion

Engaging in vigorous-intensity levels of physical activity and possessing a higher level of positive thinking were associated with the Global PSQI sleep quality among the college students. This study suggests that integrating vigorous-intensity physical activity and exercises into daily routine and maintaining high levels of positive thinking play essential roles for having a good sleep quality among college students. As participants in this study appeared to minimally participate in physical activity, this study further suggests that PE teachers should use appropriate instructional strategies to effectively engage students in vigorous-intensity physical activity tasks as much as possible during a regular PE lesson regardless of what specific PE content. Given the limited PE class offered per week, the PE teachers should encourage their students to engage in physical activity outside of the PE class by giving specific bonus grading points to the students who did so. In addition, this study suggests that a future intervention program should invest effective intervention strategies for helping college students develop and maintain positive thinking mindsets.

## Data Availability

The datasets generated during and/or analyzed during the current study are available from the corresponding author on reasonable request.
